# Impact of *Limosilactobacillus fermentum* probiotic treatment on gut microbiota composition in sahiwal calves with rotavirus diarrhea: A 16S metagenomic analysis study”

**DOI:** 10.1186/s12866-024-03254-z

**Published:** 2024-04-04

**Authors:** Nadeem Murtaza, Muhammad Nawaz, Tahir Yaqub, Asim Khalid Mehmood

**Affiliations:** 1 Institute of Microbiology, University of Veterinary and Animal Sciences, Lahore, 54000 Pakistan; 2 Department of Veterinary Surgery and Pet Sciences, University of Veterinary and Animal Sciences, Lahore, 54000 Pakistan

**Keywords:** Rotavirus, Probiotic, Diarrhea, Shannon index, 16S metagenomics

## Abstract

**Background:**

Diarrhea poses a major threat to bovine calves leading to mortality and economic losses. Among the causes of calf diarrhea, bovine rotavirus is a major etiological agent and may result in dysbiosis of gut microbiota. The current study was designed to investigate the effect of probiotic *Limosilactobacillus fermentum* (Accession No.OR504458) on the microbial composition of rotavirus-infected calves using 16S metagenomic analysis technique. Screening of rotavirus infection in calves below one month of age was done through clinical signs and Reverse Transcriptase PCR. The healthy calves (*n* = 10) were taken as control while the infected calves (*n* = 10) before treatment was designated as diarrheal group were treated with Probiotic for 5 days. All the calves were screened for the presence of rotavirus infection on each day and fecal scoring was done to assess the fecal consistency. Infected calves after treatment were designated as recovered group. Fecal samples from healthy, recovered and diarrheal (infected calves before sampling) were processed for DNA extraction while four samples from each group were processed for 16S metagenomic analysis using Illumina sequencing technique and analyzed via QIIME 2.

**Results:**

The results show that *Firmicutes* were more abundant in the healthy and recovered group than in the diarrheal group. At the same time *Proteobacteria* was higher in abundance in the diarrheal group. Order *Oscillospirales* dominated healthy and recovered calves and *Enterobacterials* dominated the diarrheal group. Alpha diversity indices show that diversity indices based on richness were higher in the healthy group and lower in the diarrheal group while a mixed pattern of clustering between diarrheal and recovered groups samples in PCA plots based on beta diversity indices was observed.

**Conclusion:**

It is concluded that probiotic *Limosilactobacillus Fermentum* N-30 ameliorate the dysbiosis caused by rotavirus diarrhea and may be used to prevent diarrhea in pre-weaned calves after further exploration.

**Supplementary Information:**

The online version contains supplementary material available at 10.1186/s12866-024-03254-z.

## Background

Diarrhea results in heavy losses in terms of mortality and economics and is regarded as a major intestinal disease in calves [1]. It has been reported that up to 57% of mortality in calves especially in calves of less than one month of age occurs due to diarrhea [2]. Different infectious agents are responsible for causing diarrhea. Among the viral agents rotavirus is considered a major etiological agent causing acute diarrhea in cattle calves globally [3, 4],

Bovine rotavirus infects calves below 2 weeks of age and takes 12–24 h (incubation period) to produce diarrhea with excretion of virus in affected calves occuring within 5–7 days via feces. The excretion of virus thus predisposes the environment to contamination and a source of spreading the infection to uninfected calves [5]. Diarrhea appears when the destruction of epithelial cells of villi occurs. Vasoactive components like histamine and viral enterotoxin contribute to malabsorption and ultimately lead to diarrhea [6]. The pathogens responsible for causing intestinal infections are believed to result in disturbing the normal microbial composition of the intestinal tract, which can further worsen or intensify the symptoms of diarrhea. Infection with rotavirus has been proven to affect the microbiota of the intestine and results in its alteration in terms of bacterial taxa abundance, diversity, homogeneity and overall configuration [7]. The normal balanced gut microbiota impedes the invasion of the intestine by pathogens and helps in strengthening the immune system in the early days of life via interaction of the antigens with the defense system of the body. Dysbiosis in intestinal microbiota may promote inflammation in the intestine, alteration in immune response and affect the utilization of nutrients by intestinal flora and their competition with pathogens for these nutrients [8].

Rotavirus is a viral disease and it has no specific treatment and it is majorly treated symptomatically by providing supportive options including fluid therapy to recover fluid losses and restore electrolyte imbalance [9]. Different studies have reported that restoring normal gastrointestinal microbiota is an effective prophylactic and therapeutic strategy for infections of GIT [10, 11]. Restoring the imbalance of microbiota through fecal transplantation from healthy animals has proven successful in reducing diarrhea in calves [12]. Beneficial living microorganisms known as probiotics are known for their use as therapeutic and prophylactic purposes in gastroenteritis and exerting beneficial effects on improving gut health, enhancing immunity [13, 14] and improving and regulating the balance of gut microbiota [15, 16]. Rumen ecosystem improves after adding probiotics in feed due to the enzymes produced by these bacteria and enhancement in beneficial bacterial communities growth and function leads to stabilization of normal GIT microbiota [17]. Use of multispecies probiotics has shown promising results in shortening the duration of diarrhea in calves [18]. Probiotic bacterial strains of *Bifidobacterium* and *Lactobacillus* are found to have positive impact in combating rotavirus infection [19, 20]. The findings of a study also show that use of a milk replacer having a multispecies probiotic added lessens the intensity of BRV(Bovine RotaVirus) induced diarrhea and reduces the intestinal lesions due to enteritis [21], Therefore use of probiotics may be a possible therapeutic or prophylactic option in controlling diarrhea induced by viral enteropathogens such as BRV by restoring normal gut microbial commmunaties. The current study is designed to study the effect of single species probiotic (bovine intestinal origin) on fecal microbial composition and to check the differences in fecal microbiota in healthy, rotavirus-infected calves and recovered calves after administering probiotic. The variations detected in study groups will help in identifying the signature microbes that are known to increase or decrease with the rotavirus infection and the effect of probiotic on the microbiota of recovered calves. The findings will also help in selecting beneficial bacteria that are identified in healthy and recovered calves for future use as a candidate for use as probiotic in controlling the disease.

## Methods

### Sample collection and processing

The sampling for this study was carried out at Livestock Production and Research Institute (LPRI) Bahadar Nagar, 56,300 Okara- Pakistan. The calves used in this research was of Sahiwal breed and below one month of age reared under similar husbandry conditions and fed milk through buckets (Supplementary Table. S1) At the first step a total of 20 calves were enrolled including ten healthy calves and ten calves having diarrhea and found positive for rotavirus infection. Screening of the calves for the presence of rotavirus infection was done through visual inspection of clinical signs (fecal scoring) [22]and confirmation through Reverse Transcription Polymerase Chain Reaction (RT-PCR) from fecal samples. Viral RNA extraction was done from fecal samples using GeneJet RNA purification kit (Thermo, USA) followed by cDNA synthesis through Thermo Scientific Revert Aid First Strand cDNA Synthesis kit (Thermo, USA) following the guidelines outlined in the manufacturer instructions manual [23]. Bovine rotavirus was confirmed by targeting the VP6 gene for amplification using specific primers [24]. Healthy calves were taken as control while calves having rotavirus infection were named as as diarrheal group before treatment and were given an oral dose (1 × 10^8^CFU/ml) of Probiotic (*Limosilactobacillus fermentum* N-30, NCBI Accession No.OR504458) for 5 days. The probiotic strain (*Limosilactobacillus fermentum* N-30 isolated from Sahiwal cattle calves feces and already characterized and tested for probiotic properties) was taken from Probiotic research laboratory, Institute of Microbiology, UVAS Lahore. The diarrheal group was designated as recovered group after treatment. No medicines were given to the calves having diarrhea during the course of experiment except probiotics. Furthermore the fecal scoring (Supplementary Table. S1) and screening using RT PCR was daily done. The treatment was continued till the diarrhea completely subsided and fecal samples were found negative for rotavirus. Fecal sampling for metagenomic analysis was done at two instances i.e. before starting treatment from healthy group and diarrheal group and after the treatment from recovered calves. 20gm fecal sample from each calf was collected in air tight specimen collection container and transportation was done at controlled temperature (4 °C). Samples were kept at -20 °C before processing. All the laboratory work was carried out at Probiotic Research Laboratory, Institute of Microbiology, University of Veterinary and Animal Sciences (UVAS), Lahore.

### Bacterial DNA extraction and quality check

Processing of the samples was carried out within 24 h of collection. Initially all the samples were processed for DNA extraction using the kit method (Fast DNASpin Kit (MP Biomedicals) following the instructions of the manufacturer [25]. Two methods were used to assess the quality and quantity of extracted DNA. Spectrophotometric method, in which the DNA(quantity and quality) was assessed through Multiskan Sky microplate spectrophotometer (Thermoscientific, Waltham, MA, USA). Samples passing the criteria of having O.D in the range of 1.6–1.9 at 260/280nm were further subjected to16S rDNA amplification using set of universal primer i.e.8FLP and XB4 primers (AGTTTGATCCTGGCTCAG, GTGTGTACAAGGCCCGGGAAC) as described previously [26]. The amplified products were checked for the presence of band using 1.5% agarose gel. Among all DNA samples, 12 samples were processed for metagenomic analysis. Four samples were selected from each of the healthy, diarrheal and recovered group for metagenomic analysis. Samples were properly labeled, packed and dispatched to Macrogen (Seoul, South Korea) for further processing for metagenomic sequencing targeting 16S rRNA gene.

### Sequencing and microbiome analysis

Briefly, target regions (V3 and V4) for 16S rDNA were amplified with a set of primers including 341F (5′-CCTAYGGGRBGCASCAG-3′) and 806R (5′-GGACTACNNGGGTATCTAAT-3′) utilizing specific barcode [17]. MiSeq (2 × 300 bp) platform was used for sequencing through the Illumina DNA Prep Kit (Illumina, San Diego, CA, USA).The Illumina 16S Metagenomic Sequencing Library Preparation Part #15044223 Rev. B document’s instructions were adopted during the library preparation step, as stated in the protocol.

Bioinformatics analysis was performed using QIIME 2(Version 2.2020.6) [27]. Raw reads (pair end) were imported to QIIME pipeline through import command mentioned in the guidelines available in QIIME 2 online tutorial. The refinement of overall sequence quality was achieved by removing noisy data through DADA2’s denoise-paired approach [28]. A 300 bp criteria was selected for trimming and sequences within this criterion were further processed by excluding longer and chimeric sequences. The operational taxonomic units (OTU’s)based classification of bacteria at different taxonomic levels was performed with SILVA database used as a reference and with a threshold of 99% similarity [29].Venn diagrams were used to show the similar and distinct taxa at different levels. Frequency tables were generated in QIIME 2 and bacterial taxonomic composition was graphically presented using taxa barplots and heat maps.

### Statistical analysis

Diversity within the samples was assessed with alpha diversity indices [30] specifically Shannon, Simpson, Chao 1 and Observed species and analyzed using the non-parametric Kruskal Wallis test, while between the samples diversity was assessed with beta diversity indices such as weighted UniFrac, un-weighted UniFrac [31, 32], Jaccard and Bray–Curtis dissimilarity indices [33, 34]and analyzed using PERMENOVA. The variations among the bacterial communities at different levelswere assessed using ANOVA (Analysis of variance) though GraphPad Prism 8.0.1. (GraphPad Software, San Diego, CA, USA).

## Results

### Clinical signs and RT PCR results in healthy diarrheal and recovered groups

The clinical finding and RT PCR results are presented in the supplementary table. S1 indicates that healthy group calves were negative for RT-PCR during the course of experiment. whereas according to fecal scoring of calves treated with probiotic had a fecal scoring of 3, which represents watery consistency of the feces on first day after probiotic treatment and rotavirus was detected in their fecal samples. Improvement in fecal score was observed on 2^nd^ and 3^rd^ day of probiotic administration and all the calves fecal samples were of normal consistency on 4^th^ day and rotavirus was not detected in 4 out of 10 calves. On 5^th^ day all the calves were found negative for rotavirus infection both symptomatically and through RT-PCR.

### Bacterial diversity and taxonomic picture at different levels in fecal microbiota of calves

The 16S RNA gene base metagenomic analysis of fecal samples (*n* = 4) from each of three groups of calves including healthy, diarrheal and recovered groups revealed a total of 34,835 OTU’s with a range of 1384–5088. The OTU’s found in healthy calves group were 15,564 with a range of 2714–5088, while in diarrheal calves group the lowest OTU’s were detected amongst the three groups i.e. 9545 with a range of 1384 to 2938. The total reads detected in recovered calves were 10,726 having a range of 1518–3488.

#### Distinct and shared bacterial taxa at different taxonomic levels

**Fig. 1:**
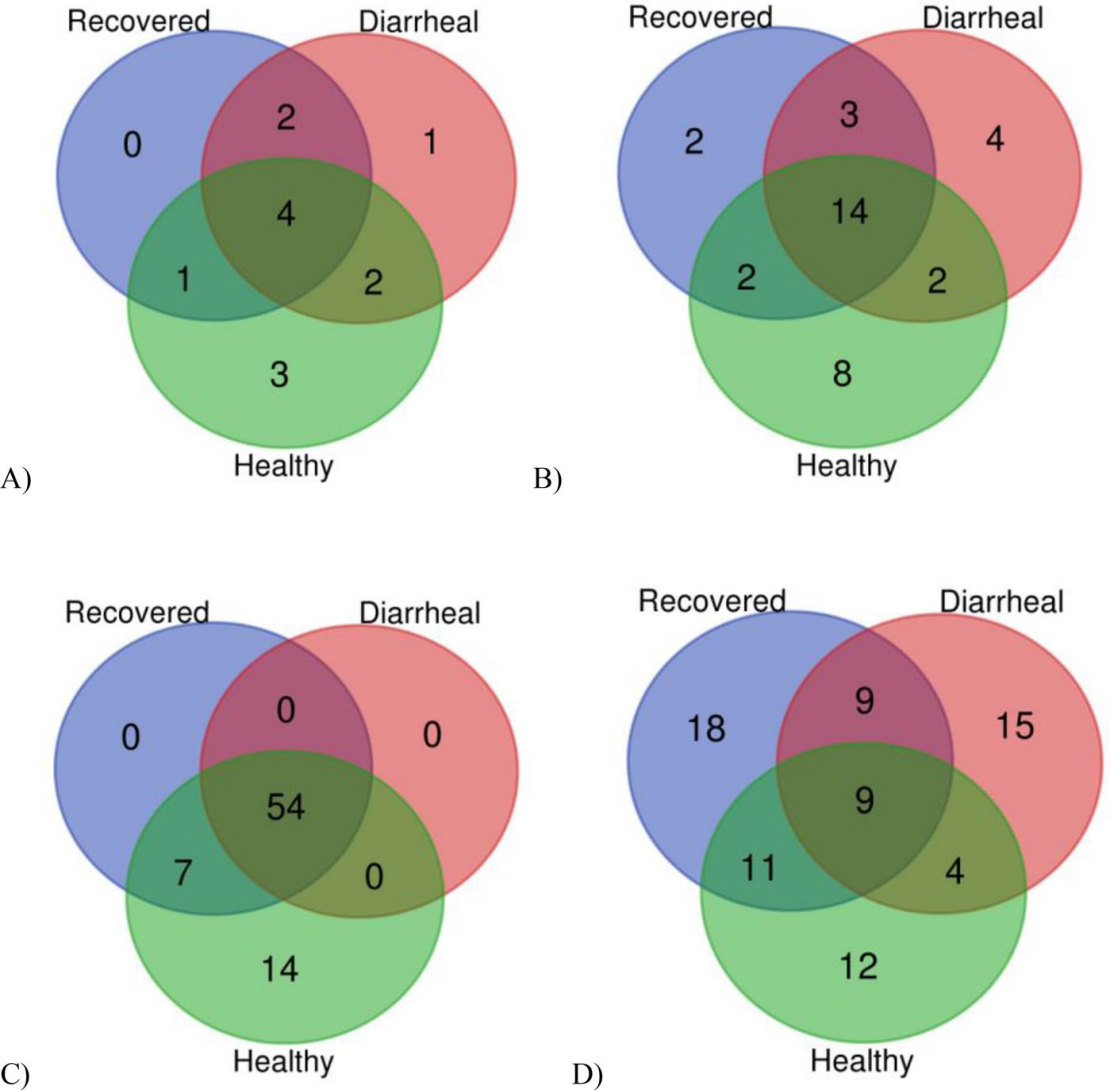
Shared and distinct taxa observed in fecal microbiota in three groups at (A) Phylum, (B) Order, (C) Genus and (D) Species Level

Fecal microbiota analysis shows that there were collectively 13 phyla that were present within all three groups, with four phyla being common to all of them. Common phyla among all groups included *Bacteroidota, Desulfobacterota, Firmicutes and Proteobacteria*. The number of detected phyla varied in fecal microbiota of recovered (7), healthy (10) and diarrheal group (9). No distinct phyla were detected in recovered group while 3 (*Actinobacteriota, Campilobacterota, Planctomycetota*) and 1 (*Verrucomicrobiota*) distinct phyla were detected in healthy and diarrheal group respectively. Diarrheal group shared 2 phyla each with healthy and recovered group while both healthy and recovered group (Fig. 1A).The total orders that were detected in all the groups were 35 in which distinct orders noted in healthy calves group were 8 and in diarrheal calves group were 4 and only 2 distinct orders were detected in recovered calves group samples as illustrated in Fig. 1B. 14 orders were shared amongst all groups, 3 orders were shared between recovered and diarrheal groups. In contrast, healthy group shared 2 orders with each of the other two groups. A total of 76 genera were detected in all groups, among those diarrheal group contained only 54 genera which were shared with the other two groups and no distinct genera was detected in this group. 14 distinct genera in the healthy calves group was detected while it shared 7 genera with recovered calves group making a total of 75 genera in this group. while 1 genus was distinct in recovered calves group among 62 genera as illustrated in Fig. 1C. The species level analysis resulted in the detection of 78 species distributed among the groups in which 18 distinct species were detected in the recovered calves group, 15 in the diarrheal calves group and 12 in the healthy calves group as represented in Fig. 1D. Nine species were shared between all the groups. While the healthy calves group and the recovered calves group shared 11 species, 9 species were shared between recovered and diarrheal calves group and 4 species between diarrheal and healthy calves group (Fig. 1D).

#### Differences in bacterial taxonomic composition at different levels

**Fig. 2:**
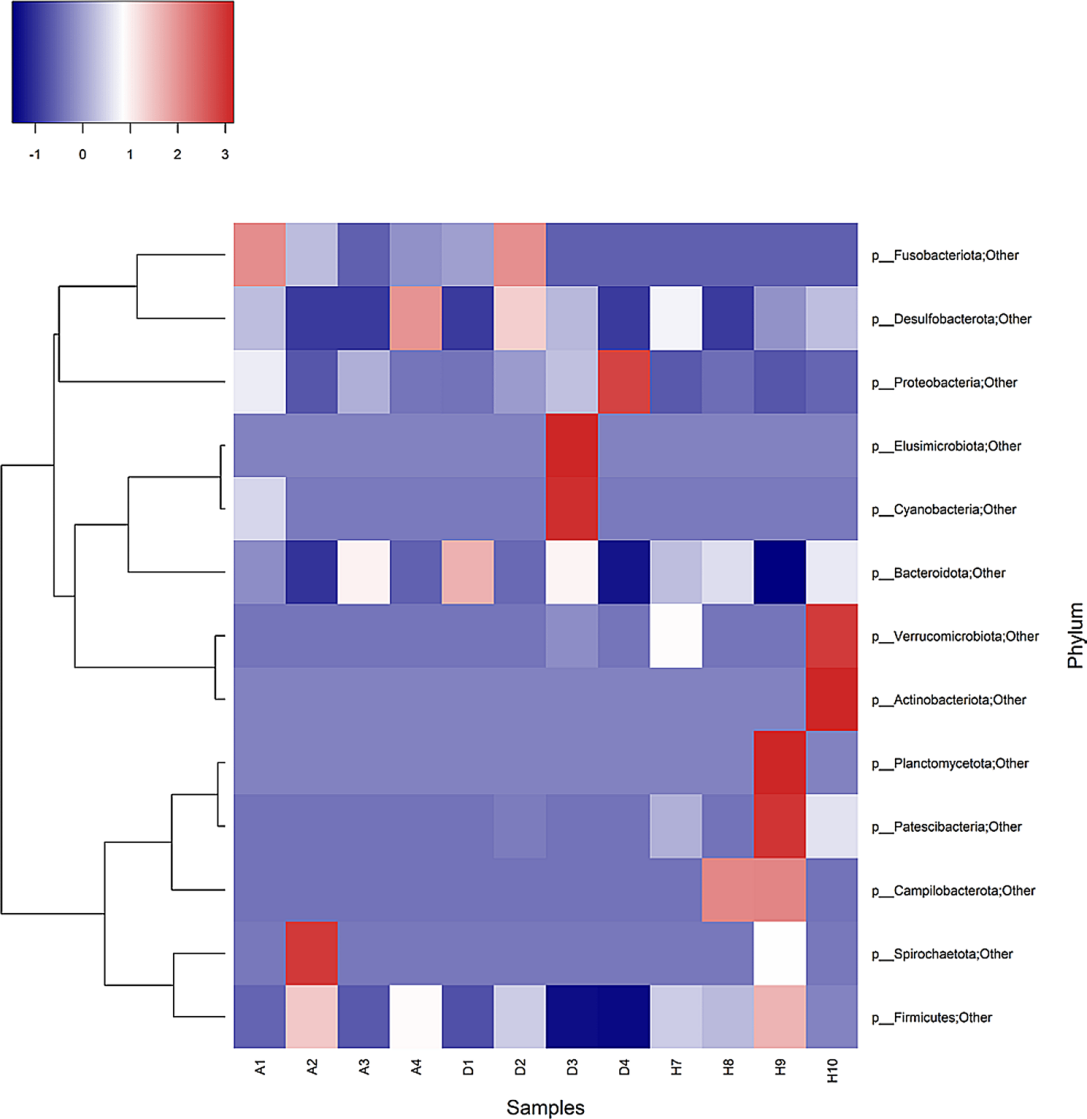
Heat map Sample wise distribution of phyla in fecal microbiota Samples A1-A4 represent recovered calves group, D1-D4 represent diarrheic calves group and H7-H10 represent healthy calves group

**Fig. 3:**
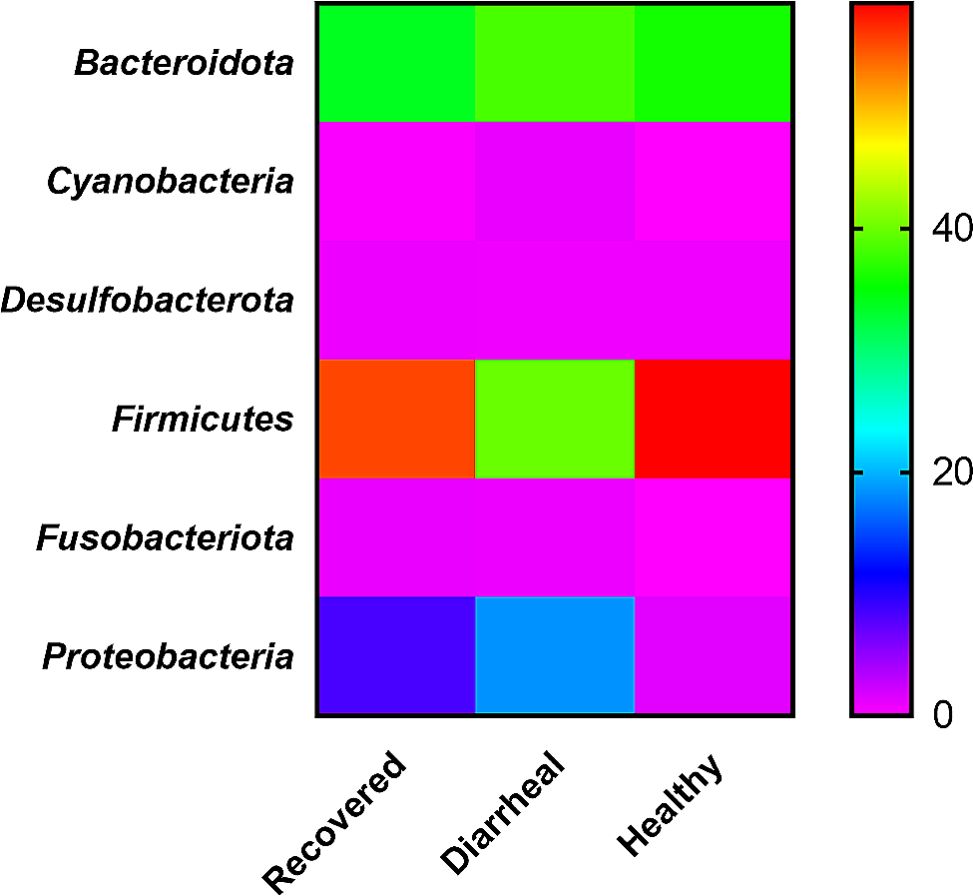
Graphical(heat map based) group wise (healthy, diarrheal and recovered calves) comparison of abundant phyla in fecal microbiota. Recovered group (*n* = 4), Diarrheal group (*n* = 4) and Healthy group (*n* = 4)

A diverse microbiota was detected at phylum level in fecal samples of all the three groups (Fig. 2). *Firmicutes, Bacteroidota* and *Proteobacteria* were the major phyla in fecal microbiota in all the groups. The recovered calves group and healthy calves group was dominated by *Firmicutes* with percentage abundance of 55.30% and 58.50% respectively followed by *Bacteroidota* with percentage abundance of 33.66% and 35.90% in recovered calves and healthy calves respectively (Table 1). The diarrheic group showed almost similar percentage abundance of *Firmicutes* (39.86%) and *Bacteroidota* (38.40%) as represented in Table 1; Fig. 3. The *Proteobacteria* phylum abundance was high in the diarrheal group (18.39%) as compared to healthy calves group (1.31%) and recovered calves group (8.38%). In addition to these phyla, *Desulfobacterota* (0.79%) and *Fusobacteriota* (0.99%) were highest in recovered group while the diarrheic group contained higher *Cyanobacteria* (0.97%) compared to other groups.

**Table 1:** Abundance (%) of bacterial taxa in fecal samples of cattle calves of first week age. Recovered group (*n* = 4), Diarrheal group (*n* = 4) and Healthy group (*n* = 4)

Taxonomic Level		Recovered (%)	Diarrheal (%)	Healthy (%)
**Phylum**	***Bacteroidota***	33.66	38.40	35.90
	***Cyanobacteria***	0.24	0.97	0.00
	***Desulfobacterota***	0.79	0.66	0.71
	***Firmicutes***	55.30	39.86	58.50
	***Fusobacteriota***	0.99	0.81	0.00
	***Proteobacteria***	8.38	18.39	1.31
**Order**	***Bacteroidales***	33.58	37.84	35.59
	***Christensenellales***	0.98	0.00	5.90
	***Clostridia_UCG-014***	6.65	1.89	11.93
	***Clostridia_vadinBB60_group***	1.53	1.48	0.46
	***Desulfovibrionales***	0.79	0.66	0.71
	***Enterobacterales***	7.44	14.18	0.00
	***Erysipelotrichales***	4.77	1.13	2.08
	***Fusobacteriales***	0.99	0.81	0.00
	***Lachnospirales***	12.92	12.21	9.10
	***Lactobacillales***	6.98	10.51	1.08
	***Oscillospirales***	17.63	7.65	22.21
**Genus**	***[Eubacterium]coprostanoligenes***	0.00	0.09	4.58
	***Alloprevotella***	1.94	3.28	2.79
	***Bacteroidales***	0.24	0.10	0.06
	***Bacteroides***	19.31	17.58	17.41
	***Christensenellaceae***	1.01	0.00	6.59
	***Clostridia_UCG-014***	7.00	1.90	12.72
	***Desulfovibrio***	0.88	0.69	0.77
	***Fusobacterium***	1.05	0.84	0.00
	***Lactobacillus***	6.77	9.57	1.14
	***Rikenellaceae***	0.92	1.09	5.47
	***Ruminococcus***	1.52	1.74	0.33
**Species**	***Bacteroides massiliensis***	4.15	0.00	1.78
	***Bacteroides nordii***	0.96	0.91	0.00
	***Bacteroides uniformis***	4.95	1.34	0.00
	***Bacteroides vulgates***	1.96	9.36	0.86
	***Butyricicoccus pullicaecorum***	1.33	0.00	0.62
	***Fusobacterium mortiferum***	0.80	1.95	0.00
	***gut metagenome***	3.85	5.58	8.23
	***Parabacteroides chinchilla***	0.40	0.17	0.03
	***uncultured bacterium***	30.55	26.13	51.87
	***uncultured Bacteroidales***	0.00	0.00	2.42
	***uncultured Clostridiales***	2.13	0.00	1.51
	***uncultured Firmicutes***	1.37	0.70	0.00
	***uncultured Lachnospiraceae***	0.27	0.00	3.15

**Fig. 4:**
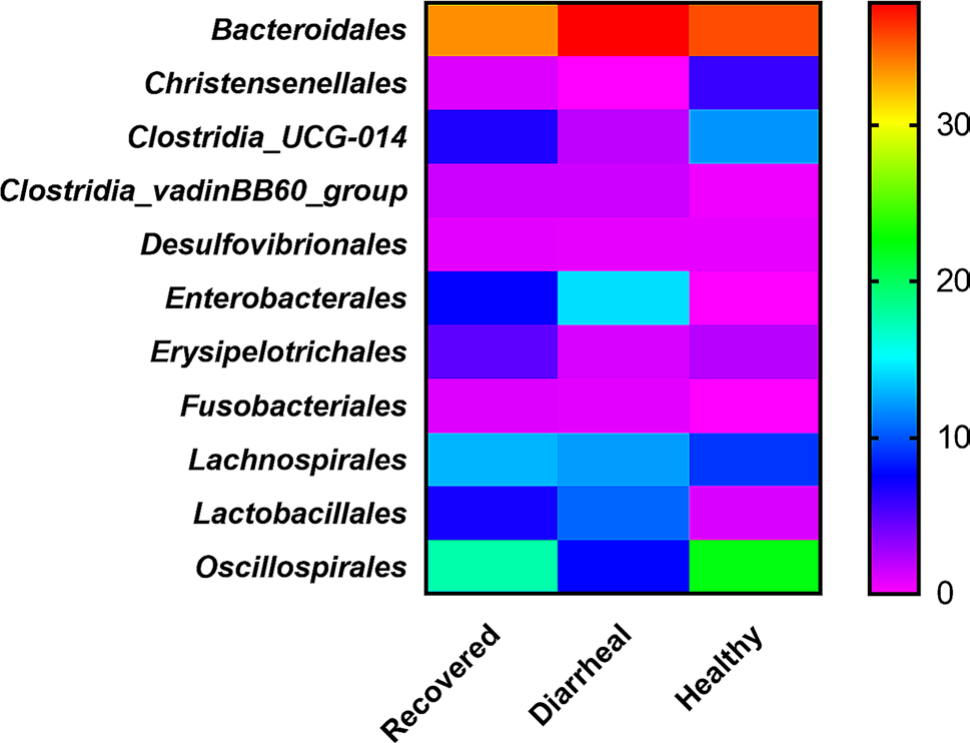
Graphical (heat map based) group wise (healthy, diarrheal and recovered calves) comparison of abundant orders in fecal microbiota. Recovered group (*n* = 4), Diarrheal group (*n* = 4) and Healthy group (*n* = 4)

The major order detected in all the groups was *Bacteroidales* with high abundance observed in diarrheal calves group (37.84%) followed by recovered calves group (33.58%) and least in healthy group (35.59%) as revealed in Fig. 4. The 2nd abundant order in diarrheal calves group recorded was *Enterobacteriales* (14.18%) while *Oscillospirales* was in healthy (22.21%) and recovered group (17.63%). In addition recovered calves group samples other orders including *Lanchospirales*, (12.92%), *Clostridia_UCG_014*(6.65%) *Enterobacteriales* (7.44%) and *Lactobacillales* (6.98%) were identified. Orders including *Lanchospirales*(12.20%), *Oscillospirales* (7.65%) and *Clostridia_UCG_014* (1.89%) having more than 1.5% abundance were also recorded in diarrheal calves group. *Clostridia_UCG_014* (11.93%), *Lanchospirales* (9.10%) and *Christenellales* (5.90%) were the other 3 major orders observed in healthy calves group (Table 1).

**Fig. 5:**
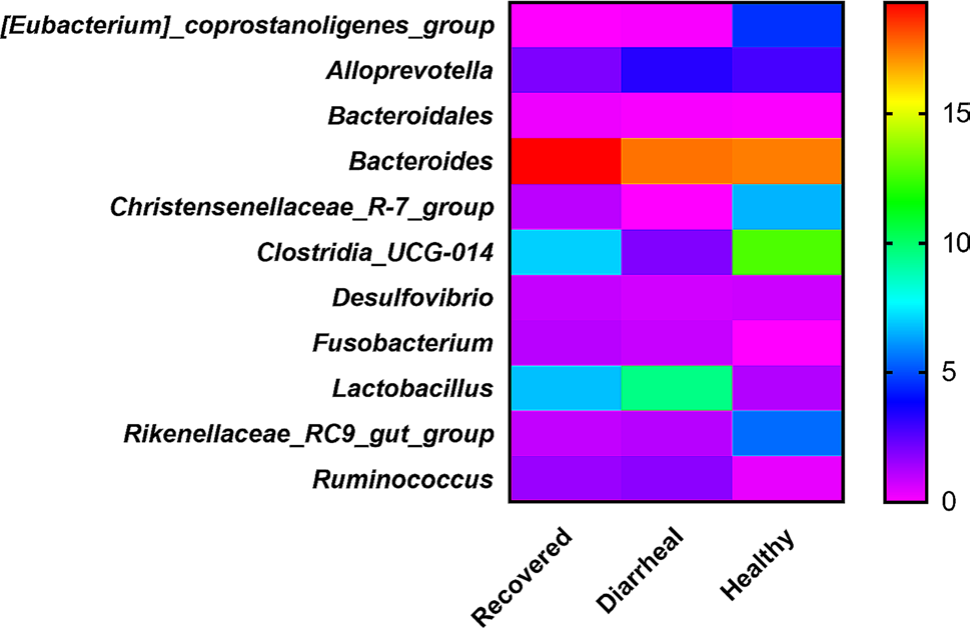
Graphical (heat map based) group wise (healthy, diarrheal and recovered calves) comparison of abundant genera in fecal microbiota. Recovered group (*n* = 4), Diarrheal group(*n* = 4) and Healthy group (*n* = 4)

The percentage abundance of different genera is presented in Table 1, in which the *Bacteroides* representation in all the groups is high, as shown by its percentage abundance in recovered calves group (19.31%), followed by diarrheal calves group (17.58%) and healthy calves group (17.41%). Genus *Clostridia_UCG_014* (7%) represented the 2nd most abundant genera in recovered calves group. In comparison *Lactobacillus* (6.77%) ranked 3^rd^ in terms of abundance as shown in Fig. 5. In the diarrheal calves group *Lactobacillus* was at 2^nd^ position in terms of abundance (9.57%) followed by *Alloprevotella* (3.28%). The abundance of *Lactobacillus* in the healthy group (1.14%) was very low as compared to the other two groups. *Bacteroides* (17.41%) was the most abundant genus in the healthy group followed by *clostridia_UCG_014* as the 2^nd^ most abundant (12.72%) genus and *Christensenellaceae* being 3^rd^ in terms of abundance (6.59%) as shown in Fig. 5. Other genera were also detected in three groups but their abundance was low. *Bacteroidales*, *Christensenellaceae, Desulfovibrio, Fusobacterium, Rikenellaceae* and *Ruminococcus* were detected in low abundance in the recovered calves group. In the diarrheal calves group *[Eubacterium]coprostanoligenes, Bacteroidales, Desulfovibrio, Fusobacterium, Rikenellaceae* and *Ruminococcus* were detected but in low abundance. While in the healthy calves group low abundant genera that were detected included *Rikenellaceae, [Eubacterium]coprostanoligenes, Bacteroidales, Desulfovibrio, Fusobacterium* and *Ruminococcus*d(Table 1).

**Fig. 6:**
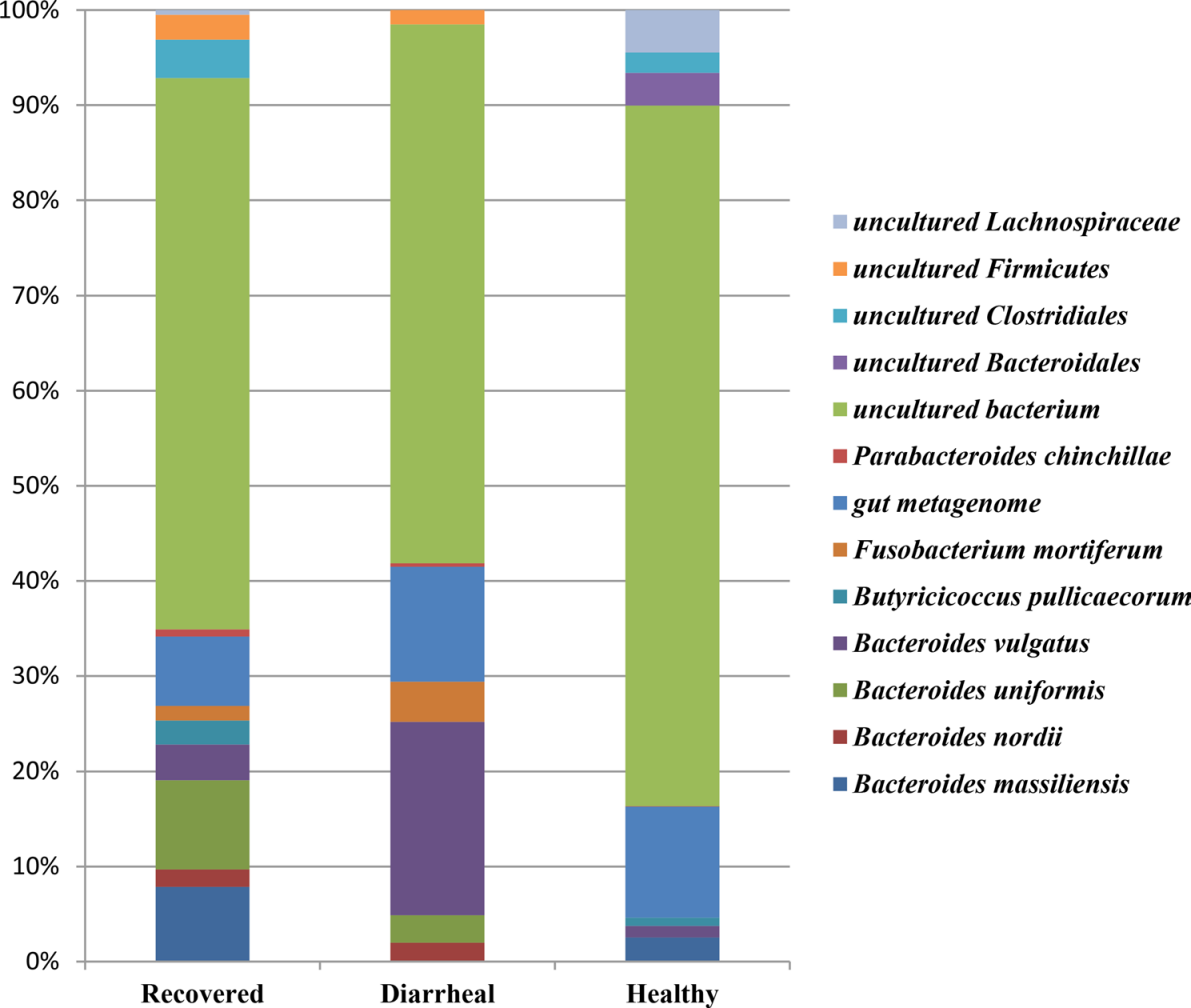
Taxbar plot showing the different abundant species in recovered, diarrheal and healthy cattle calves groups. Recovered group (*n* = 4), Diarrheal group (*n* = 4) and Healthy group (*n* = 4)

The different abundant species detected in samples of the three groups are represented in Fig. 6. Species level distribution of fecal microbiota resulted in most of the species being assigned into unculturable and unassigned species. Some species that were detected in recovered calves group with varying abundance included *Bacteroides uniformis* (4.95%), *Bacteroides massiliensis* (4.15%), *Bacteroides vulgates* (1.96%), *Butyricicoccus pullicaecorum* (1.33%), *Bacteroides nordii* (0.96%), *Fusobacterium mortiferum* (0.80%) and *Parabacteroides chinchilla* (0.40%) as shown in Fig. 6. *Bacteroides vulgates* (9.36%), *Fusobacterium mortiferum* (1.95%), *Bacteroides uniformis* (1.34%), *Bacteroides nordii *(0.91%), and *Parabacteroides chinchilla* (0.17%) were detected in diarrheal group. The healthy group included *Bacteroides massiliensis* (1.78%), *Bacteroides vulgates* (0.86%), *Butyricicoccus pullicaecorum* (0.62%) and *Parabacteroides chinchilla* (0.03%) as shown in Table 1.

### Diversity analysis

#### Alpha diversity

**Fig. 7:**
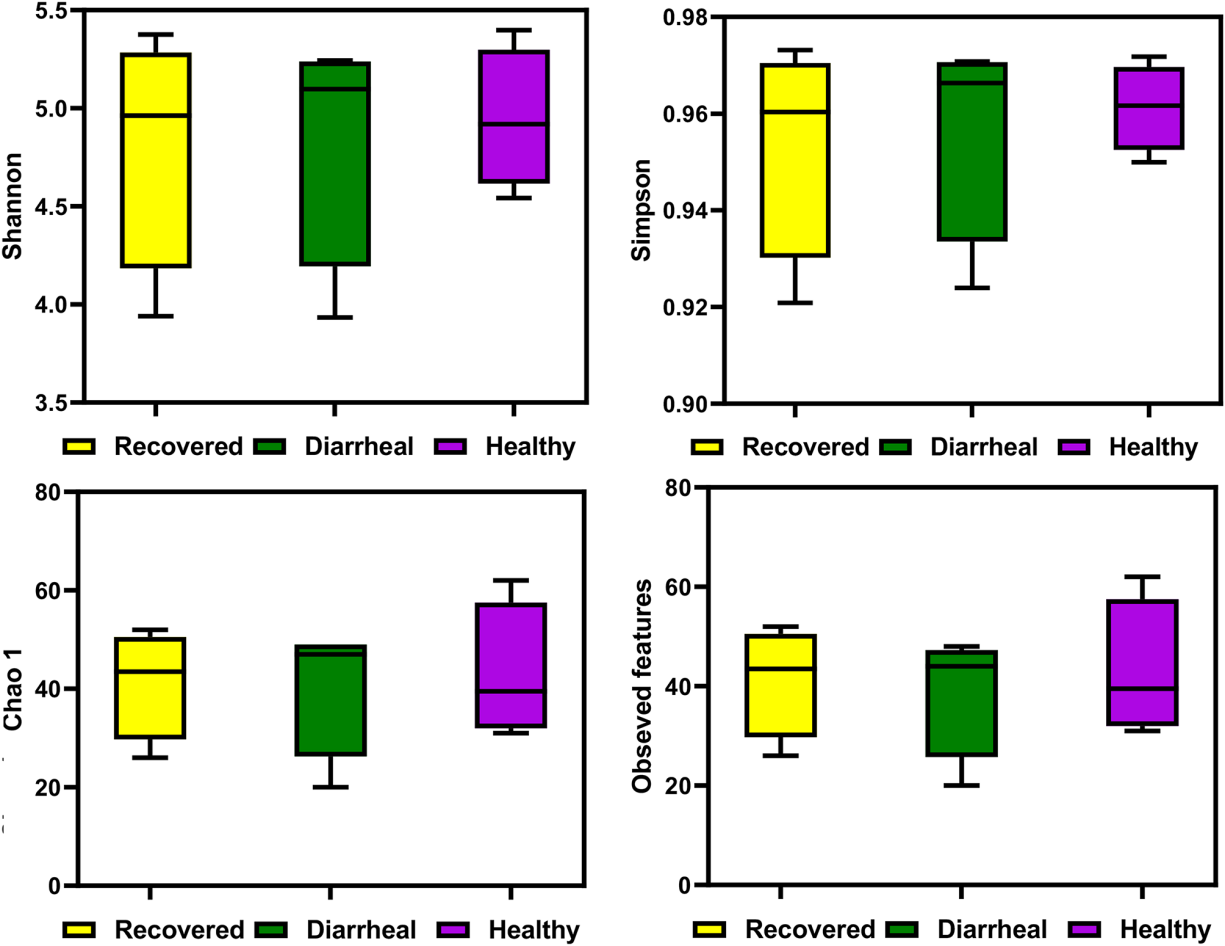
Alpha diversity indices across groups

Microbiota associated with healthy calves was found to be more diverse as depicted by different diversity indices. Shannon diversity index in healthy calves group was found to be the highest and represented as Mean ± SEM was 4.94 ± 0.17, while lowest diversity was detected in the recovered calves group (4.81 ± 0.30) and its value in the diarrheal group was 4.84 ± 0.30 as shown in Fig. 7. The Simpson diversity index was found to be highest in the healthy calves group (0.96 ± 0.00) while in the other two groups its value was same (0.95 ± 0.01) as given in Table 2. Chao1 and observed features indices showed that diversity was high in healthy group(43 ± 6.89) followed by recovered calves group(41.25 ± 5.55) and lowest in diarrheal calves group with values of 40.75 ± 6.98 for Chao 1 and 39 ± 6.41 for observed features. There was no significant difference observed among the different groups in any diversity index. However, the overall pattern shows that the samples of healthy group comprised of diverse microbiota and microbial diversity decreased in recovered and diarrheal group as represented by different indices (Fig. 7).

**Table 2:** Alpha diversity indices in different groups of calves fecal microbiota

Indices	Recovered	Diarrheal	Healthy
	Mean ± SEM	Mean ± SEM	Mean ± SEM
**Shannon**	4.81 ± 0.30	4.84 ± 0.30	4.94 ± 0.17
**Simpson**	0.95 ± 0.01	0.95 ± 0.01	0.96 ± 0.00
**Chao 1**	41.25 ± 5.55	40.75 ± 6.98	43 ± 6.89
**Observed features**	41.25 ± 5.55	39 ± 6.41	43 ± 6.89

#### Beta diversity

**Fig. 8:**
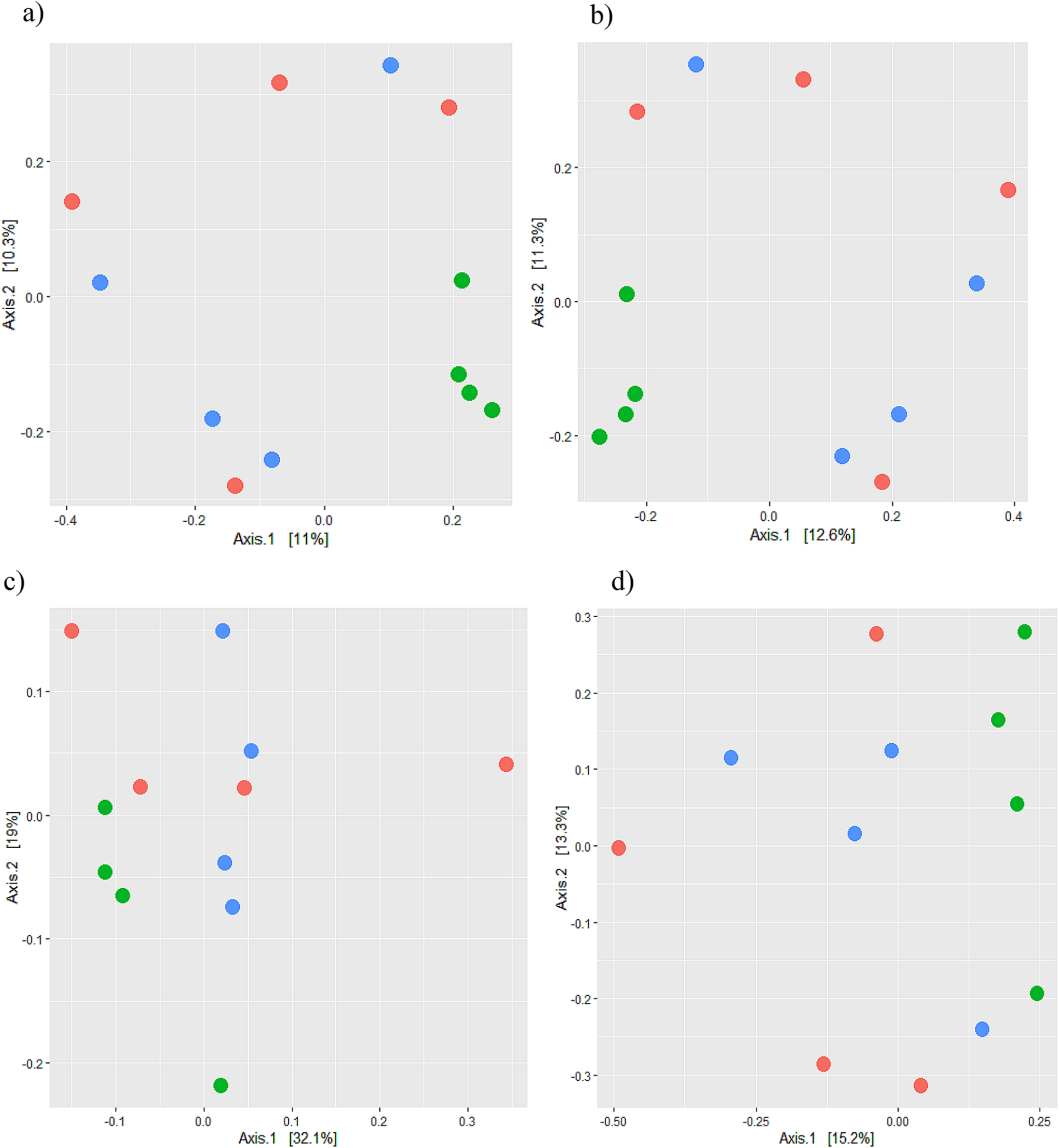
Diversity indices (a. Jaccard b. Bray Curtis c) Weighted UniFrac distance and d) Unweighted UniFrac distance) representation through PCA plots. Different colors represents samples of different groups (Red: Diarrheal, Green: Healthy and Blue: recovered)

The bacterial diversity PCA plot-based pictures (Fig. 8) in terms of non-phylogenetic diversity indices (Jaccard and Bray Curtis) shows clustering of healthy group samples separately and far from diarrheal and recovered groups, whereas recovered and diarrheal samples have clustered in mix pattern with each other. The Phylogenetic-based indices (both weighted and unweighted UniFrac distance indices show a mix pattern, in which 3 out of 4(75%) healthy samples have clustered together, whereas recovered samples have clustered along one axis. The diarrheal samples show a scatter pattern. The PCA plot of unweighted Unifrac indice shows that healthy samples have clustered along one line while recovered samples have not been clustered at one place but distance from each other is less as compared to diarrheal samples.

## Discussion

The major cause of mortality and reduced growth performance in calves at the neonatal stage is diarrhea affecting the farmers economically [2] Bovine rotavirus is considered as the most important etiological agent among infectious causes of diarrhea in bovine calves as well as humans infants [35]. Approximately 27–36% of calves are known to have rotavirus infection [36] with mortality ranging from 5 to 20% and may reach upto 80% [37]. Along with the economic impact associated with rotavirus, its zoonotic potential cannot be undermined [9]. The use of beneficial bacteria known as probiotic has shown promising results both as prophylactic and therapeutic options for various bacterial and viral infectious diseases in bovines, poultry and humans [38–41]. As normal microbiota play important role in normal physiological condition of specific niches and maintenance of homeostasis, any alteration may result in predisposing to disease conditions [42]. Restoring the normal microbial make up of gut may help in recovery from disease associated with dysbiosis in gut [6]. Probiotic may be used as an effective tool for stabilizing the disturbed gut microbiota [43, 44].

The current study was carried out to check whether indigenous probiotic isolated from bovine feces can help in improving the microbial composition of the gut especially in a local breed calves having rotavirus infection. To reduce variation similar age group calves having almost similar weight were selected and were fed and reared under similar conditions. The probiotic administration had a positive impact on improving the fecal scoring indicating reduction in diarrhea which is in agreement with the findings of a study which states that administration of probiotics having *lactobacilli* strains positively affected the fecal scoring in calves having diarrhea [45]. Upon metagenomic analysis variations in abundance at different taxonomic levels were observed across the three groups. In a study conducted on gut microbiota of pre weaned calves using 16S metagenomic approach [46], *Firmicutes* (64–82%) was found to be the major phyla in healthy calves, *Bacteroidetes* (8–24%) and *Actinobacteria* (1–12%) were among the other major detected phyla, our findings are in line with the study in terms of abundant phyla with variation in percent abundance, as *Firmicutes* (58.50%) and *Bacteroidota* (35.90%) were major phyla found in healthy calves. The higher abundance of *Proteobacteria* in calves having rotavirus infection is detected in the current study which is in line with the findings of a study wherein they have suggested that infection with rotavirus results in dysbiosis having higher abundance of *Proteobacteria* [47]. It can be seen that *Proteobacteria* tend to be higher in recovered as compared to healthy group which may be attributed to the fact that it is in stage of transition. Abundance of *Firmicutes* was higher in recovered calves as compared to diarrheal group calves which was also reported in calves recovered from bovine corona virus, other important cause of neonatal diarrhea [48].

An interesting finding in this study was that the genus *Lactobacillus* was reported to be highest in diarrheal and recovered group as compared to healthy group. Although this is an unexpected finding but a similar finding is reported in a study in fecal microbiota in neonatal calves having GIT problems of difference severity by Slanzon and Ridenhour [49] and they have suggested that overgrowth of *Lactobacillus* may serve as recuperative strategy for gut flora in diarrheic calves due to the broad spectrum nature of *Lactobacillus*.

*Christensenellales* order has been detected in higher abundance in healthy group while it was not detected in diarrheal calves fecal microbiota. Members of *Christensenella* genus have been suggested as having probiotic potential [50] but their beneficial effect on gastrointestinal system needs exploration. *Rikenellaceae* has been detected in higher abundance in healthy calves while their abundance was not appreciable in other groups as it has been suggested that it is associated with intestinal health [51] and hypothesized to be associated with carbohydrates degradation [52] yet their role in maintaining gut health needs investigation.

Next we studied diversity using different diversity indices based on diversity within and between samples. The alpha diversity based on richness such as Chao1 and observed species indicates that diversity was less within diarrheal calves samples whereas highest in healthy group which clearly indicates that diversity tends to decrease when dysbiosis occurs due to rotavirus infection which is also reported previously [7]. In a study conducted on Holstein Friesian calves, researchers suggested that dysbiosis in the gastrointestinal tract (GIT) could be attributed to the infection of calves with rotavirus. This suggestion was based on the observation of decrease in alpha diversity indices in rotavirus-infected calves compared to healthy ones [53]. Although in another study conducted on calves having diarrhea associated with different viral pathogens including rotavirus showed differences based on microbial diversity indices from healthy calves [54] yet there were some contradiction between our observations and their findings. Chao 1 and higher quantity of OTU’s were observed to be higher in healthy claves as compared to rotavirus infected calves which was not the case in our study, the possible explanation for this would be different factors are involved in shaping the microbial composition of the gut of calves such as host related factor i.e. age, breed, sex environmental factors and infectious agent related factors such as its strain and virulence [55]. Only two alpha diversity indices (Chao1 and observed features) were a little higher in recovered group as compared to diarrheal groups but lower than healthy group which indicates that bacterial imbalance in gut microbiota occurred due to rotavirus infection seems to have been towards restoration path due to treatment [48]. The community diversity base indices Shannon and Simpson indices which take both evenness and richness into account showed a different picture. Although healthy samples diversity was higher but diarrheal and recovered groups were almost similar, which may be due to the fact that due to administration of probiotic may temporarily disturb the evenness of community as Lactic acid bacteria produces lactic acid which lowers the pH and some bacterial species are pH sensitive [56]. In our study we observed clustering of healthy samples separately from other groups samples based on beta diversity indices which are in agreement with the results of jang et al [7] they reported differences in composition of gut microbiota of healthy and rotavirus infected calves. However a mixed pattern with no clear separation was observed between diarrheal and recovered group samples which is in contradiction with the findings of the study [48], in which bovine corona virus infected calves were treated fluid therapy and they observed a clear compositional differences based on beta diversity between pre and post therapy treatment samples. The differences observed may be attributed to the difference in infectious agent, treatment type and duration of treatment which was two months in that case. The overall picture suggests that there is a clear association between the presence of a disrupted gastrointestinal ecosystem (indicated by lesser diversity in calves having diarrhea) and the occurrence of rotavirus infection and administration of probiotics to rotavirus infected calves can restore the disturbed microbiota.

## Conclusions

From the findings of the study it is concluded that *Limosilactobacillus fermentum* N-30 ameliorate rotavirus infection and dysbiosis caused by it therefore this strain may be further explored and developed as a probiotic candidate for the prevention and treatment of rotavirus diarrhea in pre-weaned calves.

### Electronic supplementary material

Below is the link to the electronic supplementary material.

Supplementary Material 1

## Data Availability

The data sets generated and/or analyzed during the current study are available in the National Center for Biotechnology Information (NCBI) under Bio Project number PRJNA1035647.
