# Supplementary material for: Impact of Limosilactobacillus fermentum probiotic treatment on gut microbiota composition in sahiwal calves with rotavirus diarrhea: A 16S metagenomic analysis study”
Source: BMC Microbiol. 2024 Apr 4;24:114. doi: 10.1186/s12866-024-03254-z (PMC10993544; doi:10.1186/s12866-024-03254-z)
Supplement: Supplementary file 1 — Supplementary Material 1 [file 12866_2024_3254_MOESM1_ESM.docx]

| Group | Animal Tag ID | Weight  (Kg) | Sex  (M/F) | Age (Days) | Fecal Score** and Rota virus detection by RT-PCR | | | | |
| --- | --- | --- | --- | --- | --- | --- | --- | --- | --- |
|  |  |  |  |  | Day 1 | Day 2 | Day 3 | Day 4 | Day 5 |
| Healthy Calves | 62/22* | 26 | F | 14 | **^ND^** | | | | |
|  | 61/22* | 26.4 | F | 13 | **^ND^** | | | | |
|  | 65/22* | 26.8 | F | 14 | **^ND^** | | | | |
|  | 71/22 | 25.6 | F | 13 | **^ND^** | | | | |
|  | 73/22 | 25 | F | 13 | **^ND^** | | | | |
|  | 59/22* | 26.4 | F | 16 | **^ND^** | | | | |
|  | 74/22 | 26.6 | F | 13 | **^ND^** | | | | |
|  | 80/22 | 28 | M | 12 | **^ND^** | | | | |
|  | 84/22 | 28.4 | M | 12 | **^ND^** | | | | |
|  | 86/22 | 29 | M | 12 | **^ND^** | | | | |
| Diarrheal Calves  (Diarrhea and treated Group) | 63/22* | 26 | F | 14 | **3^D^** | **2^D^** | **1^D^** | **0^D^** | **0^ND^** |
|  | 64/22* | 26 | F | 14 | **3 ^D^** | **3 ^D^** | **2 ^D^** | **1 ^D^** | **0 ^ND^** |
|  | 66/22* | 26.6 | F | 13 | **3 ^D^** | **2 ^D^** | **1 ^D^** | **0 ^D^** | **0 ^ND^** |
|  | 70/22 | 25 | F | 13 | **3 ^D^** | **2 ^D^** | **1 ^D^** | **0 ^ND^** | **0 ^ND^** |
|  | 72/22* | 26 | F | 13 | **3 ^D^** | **2 ^D^** | **1 ^D^** | **0 ^D^** | **0 ^ND^** |
|  | 75/22 | 24.4 | F | 13 | **3 ^D^** | **2 ^D^** | **0 ^D^** | **0 ^ND^** | **0 ^ND^** |
|  | 68/22 | 24.6 | F | 14 | **3 ^D^** | **2 ^D^** | **1 ^D^** | **0 ^D^** | **0 ^ND^** |
|  | 67/22 | 29 | M | 14 | **3 ^D^** | **2 ^D^** | **1 ^D^** | **0 ^ND^** | **0 ^ND^** |
|  | 83/22 | 28.6 | M | 12 | **3 ^D^** | **2 ^D^** | **0 ^D^** | **0 ^D^** | **0 ^ND^** |
|  | 87/22 | 28 | M | 12 | **3 ^D^** | **2 ^D^** | **1 ^D^** | **0 ^ND^** | **0 ^ND^** |

Supplementary Table S1: Calves grouping, identification and fecal scoring

*Calves used for subsequent metagenomic study; ** Diarrhea was symptomatically identified, fecal consistency was scored daily (0 = normal; 1 = pasty; 2 = semi-liquid; 3 = liquid).Diarrhea duration was defined as the number of days with fecal score ≥2; ^D^rota virus detected; ^ND^rotavirus not detected
